# The OmpA-Like Protein Loa22 Is Essential for Leptospiral Virulence

**DOI:** 10.1371/journal.ppat.0030097

**Published:** 2007-07-13

**Authors:** Paula Ristow, Pascale Bourhy, Flávia Weykamp da Cruz McBride, Claudio Pereira Figueira, Michel Huerre, Patrick Ave, Isabelle Saint Girons, Albert I Ko, Mathieu Picardeau

**Affiliations:** 1 Unité de Biologie des Spirochètes, Institut Pasteur, Paris, France; 2 Instituto de Microbiologia Professor Paulo de Goes, Universidade Federal do Rio de Janeiro, Rio de Janeiro, Brazil; 3 Centro de Pesquisas Gonçalo Moniz, Fundação Oswaldo Cruz, Salvador, Brazil; 4 Unité de Recherche et d'Expertise Histotechnologie et Pathologie, Institut Pasteur, Paris, France; 5 Division of International Medicine and Infectious Disease, Weill Medical College of Cornell University, New York, New York, United States of America; Children's Hospital Boston, United States of America

## Abstract

Pathogenic mechanisms of *Leptospira interrogans,* the causal agent of leptospirosis, remain largely unknown. This is mainly due to the lack of tools for genetic manipulations of pathogenic species. In this study, we characterized a mutant obtained by insertion of the transposon *Himar1* into a gene encoding a putative lipoprotein, Loa22, which has a predicted OmpA domain based on sequence identity. The resulting mutant did not express Loa22 and was attenuated in virulence in the guinea pig and hamster models of leptospirosis, whereas the genetically complemented strain was restored in Loa22 expression and virulence. Our results show that Loa22 was expressed during host infection and exposed on the cell surface. Loa22 is therefore necessary for virulence of L. interrogans in the animal model and represents, to our knowledge, the first genetically defined virulence factor in *Leptospira* species.

## Author Summary

The spirochetes, which include medically important pathogens such as the causative agents of Lyme disease, syphilis, and leptospirosis, constitute an evolutionarily unique group of bacteria. Leptospirosis is a zoonotic disease that causes a high rate of mortality and morbidity in humans and animals throughout the world each year. The year 2007 marks the centenary of the discovery of the causative agent of leptospirosis, Leptospira interrogans. Until now, the genetic obstacles posed by leptospires (principally, the difficulties in generating targeted mutants) have hampered the identification of virulence genes. In this study, we describe an avirulent mutant in a pathogenic *Leptospira* that was obtained via disruption of *loa22,* a gene that encodes an outer membrane protein containing an OmpA domain. This mutation resulted in an avirulent mutant in the guinea pig model, and reintroduction of *loa22* into the mutant restored *Leptospira*'s ability to kill guinea pigs. Our results therefore indicate that *loa22* is a virulence determinant that is, to our knowledge, the first identified for this pathogen.

## Introduction

Leptospira interrogans is a spirochete responsible for leptospirosis. This disease, which is considered the most geographically widespread zoonosis, has emerged as a major public health problem in developing countries [1–3]. Numerous mammalian species, including rodents, excrete the pathogen in their urine and serve as reservoirs for transmission. Humans are usually infected through contact with contaminated water or soil. Leptospirosis imparts its greatest burden on poor rural farming and urban slum populations in developing countries [1–3]. More than 500,000 cases of severe leptospirosis occur each year, with a mortality rate of 5% to 20% [4]. Little is understood of *Leptospira* pathogenesis, which in turn has hampered the identification of new intervention strategies.

Leptospires are highly motile bacteria that are able to penetrate skin and mucous membranes and rapidly disseminate to other tissues shortly after infection. In susceptible hosts such as humans, systemic infection produces severe multi-organ manifestations, including jaundice, acute renal failure, and severe hemorrhage in the lungs and other organs. However, in animal reservoirs such as the domestic rat, infection produces chronic and persistent asymptomatic carriage in the renal tubules [1–3].

The virulence mechanisms, and more generally the fundamental understanding of the biology of the causative agents of leptospirosis, remain largely unknown. To date, only a few proteins have been identified as putative virulence factors. Pathogenic leptospires have been shown to express adhesins [5,6], hemolysins [7], and many lipoproteins prominent in leptospires and other spirochetes that could play a role in host–cell interactions [8]. The recent completion of the genome sequence of pathogenic *Leptospira* strains [9–11] has provided a basis for understanding the pathogenesis of leptospirosis. However, to date, the role of putative virulence factors that were identified in the genome sequence remains speculative. The lack of genetic tools to manipulate pathogenic Leptospira spp. has prevented testing of Koch's molecular postulates [12] and researchers have been unable to elucidate the role of these determinants in virulence.

We recently provided evidence of gene transfer in *L. interrogans,* which involved the transposition of a transposon of eukaryotic origin [13]. This advance has now made it possible to apply genetic approaches to the identification of virulence determinants and vaccine candidates in pathogenic Leptospira spp. In this study, we characterized a mutant of the pathogen *L. interrogans,* which we obtained by random transposon mutagenesis. This mutant exhibited transposon insertion in a gene, *loa22,* which was described by Koizumi et al. [14] as encoding for a lipoprotein (Loa22) of 22 kDa with a C-terminal OmpA domain. Previous studies suggested that this protein may play an important role in infection [14–17]. Herein, we show that the mutant *loa22* ^−^ strain is avirulent in animal models, therefore demonstrating that Loa22 is essential for in vivo infection of pathogenic leptospires.

## Results

### Disruption and Complementation of *loa22* in L. interrogans Serovar Lai

Plasmid pMSL [13] was used to deliver the spectinomycin-resistant *Himar1* transposon into L. interrogans serovar Lai strain Lai. One of the transposon mutants exhibited an insertion in a putative gene, LA0222, encoding a protein (195 amino acids in length) that was reported by Koizumi et al*.* [14] to be Loa22, a 22-kDa *Leptospira* lipoprotein with an OmpA domain; we will therefore refer to this protein henceforth as Loa22. The L. interrogans serovar Lai protein (LA0222) exhibits 99% and 96% similarity with orthologs in the pathogens L. interrogans serovar Copenhageni (LIC_10191) and *L. borgpetersenii* serovar Hardjobovis (LBL_2925 / LBJ_0158), respectively.

The protein Loa22 exhibits a bipartite structure, which includes an N-terminal domain (residues 1–77) that is unrelated to other eukaryotic or prokaryotic protein domains, followed by an OmpA domain (residues 78–186). According to SpLip [18], an algorithm for the prediction of spirochetal lipoproteins, Loa22 is a possible lipoprotein with an atypical Leu residue prior to Cys or a probable lipoprotein with a cleavage site between residues 20 and 21, as indicated by the LipoP algorithm for lipoprotein prediction in Gram-negative eubacteria [19]. C-terminal amino acid sequence analysis of Loa22 revealed that other proteins of L. interrogans (LA4337, LA3685, LA0056, LA3615, and LB328) have sequence homology with members of the OmpA family. These L. interrogans putative proteins, including Loa22, share between 46% and 59% sequence similarity in their C-terminal domain, but they have significant amino acid sequence heterogeneity in their N-terminal domains.

Because there is no replicative plasmid vector available for pathogenic *Leptospira,* we reintroduced the wild-type copy of the gene encoding Loa22 into the spectinomycin-resistant mutant strain by using a kanamycin-resistant transposon carrying *loa22* (Figure 1C). Transposition within the chromosome is random, so we identified the transposon insertion sites in several transformants and selected one strain, TK2, for further studies (Figure 1A and 1B). Enzyme-linked immunosorbent assay (ELISA) (Figure 1D) and immunoblot analysis (unpublished data) confirmed the absence of detectable Loa22 in the mutant *loa22* ^−^ strain, whereas the protein was expressed in the wild-type and complemented strains (Figure 1D). Inactivation of *L. interrogans loa22* did not affect cell morphology and motility. The wild-type, *loa22* ^−^ *,* and TK2 strains had similar cell growth kinetics in liquid Ellinghausen-McCullough-Johnson-Harris (EMJH) medium, indicating that genetic manipulation did not alter growth in vitro.

**Figure 1:**
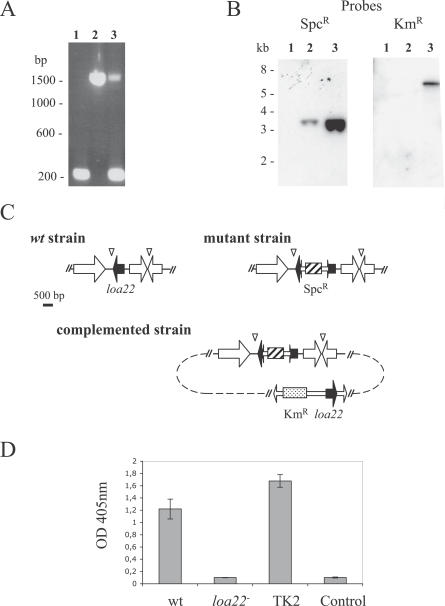
Disruption and Complementation of *loa22* in L. interrogans (A and B) Analysis of chromosomal DNA from the parental (lane 1), mutant *loa22^−^* (lane 2), and complemented TK2 strains (lane 3) by PCR with primers S1a and S1b (A) and Southern blot of EcoRI-digested DNA probed for hybridization with the spectinomycin (Spc^R^)- and kanamycin (Km^R^)-resistant cassettes (B). Primers S1a and S1b are located in the flanking sequences of the insertion site of the spectinomycin-resistant transposon into *loa22*. This analysis revealed that there was an insertion of 1.3 kb in the mutant *loa22^−^* strain and that an additional copy of *loa22* was present in the complemented strain. (C) Schematic representation of the genotype of the parental *(wt),* mutant, and complemented strains. Arrowheads in white indicate the position of EcoRI sites. (D) ELISA of plates with total bacterial antigens and Loa22 antiserum (serum dilution 1:800).

### Loa22 Is a Surface-Exposed Protein

Immunofluorescence studies found that Loa22 is a surface-exposed moiety (Figure 2). Antiserum to Loa22 labeled the surface of live wild-type and complemented TK2 strains but did not label the surface of the mutant *loa22* ^−^ strain. In control experiments, antisera to LipL32 (Figure 2) and LipL41 (unpublished data) labeled the surface of wild-type, mutant *loa22* ^−^, and TK2 strains, indicating that the labeling method was able to detect previously described surface-exposed LipL32 and LipL41 [20], but not Loa22, in the mutant *loa22* ^−^ strain. The procedure specifically detected antibodies bound to the leptospiral surface: antisera to LipL31, a previously described lipoprotein associated with the inner membrane [20] (Figure 2), and cytoplasmic heat-shock protein GroEL (unpublished data) did not label live leptospires in this procedure, although the antisera strongly labeled fixed, permeabilized leptospires (unpublished data). These results indicate that Loa22 is a surface-exposed component of the leptospiral outer membrane as previously suggested [14].

**Figure 2:**
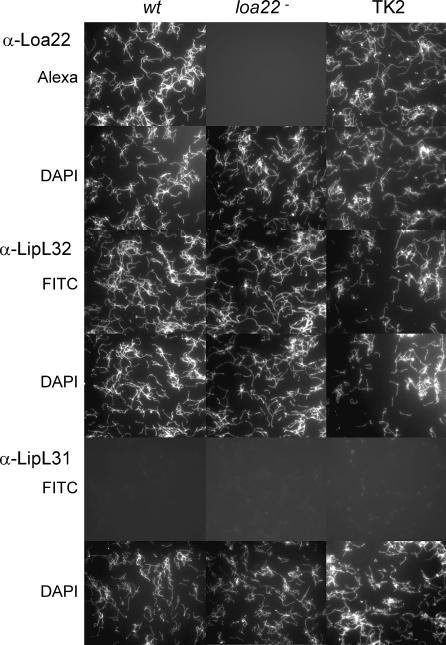
Surface Localization of Loa22 Surface immunofluorescence assay was performed with L. interrogans wild-type strain *(wt),* mutant *loa22^−^ (loa22^−^),* and mutant *loa22^−^* complemented with wild-type *loa22* (TK2). Strains were labeled with antibodies against Loa22 and the following lipoproteins: LipL32, a surface-exposed lipoprotein [20], and LipL31, a lipoprotein that is associated with the inner membrane and not surface-exposed [20]. Alexa and fluorescein isothiocyanate (FITC)–conjugated secondary antibodies were used to detect surface-bound antibodies to Loa22 and LipL32 and LipL31, respectively. A DAPI counterstain was used to document the presence of leptospires. A photomicrograph is shown from one of three representative experiments.

### Virulence of the Mutant *loa22^−^* Strain Is Attenuated in Experimental Animal Models

The guinea pig and hamster, the standard experimental models for leptospirosis [1,2], were used to evaluate the virulence of the wild-type, mutant *loa22* ^−^, and complemented strains (Table 1). In two experiments, ten of fourteen and eight of eight of the guinea pigs died when inoculated with intraperitoneal challenges of 2 × 10^8^ and 4 × 10^8^ wild-type bacteria, respectively. Infected guinea pigs developed leptospirosis with characteristic signs such as prostration and jaundice (Figure 3), and died within 4 to 6 d after the infection (Table 1).

**Table 1:**
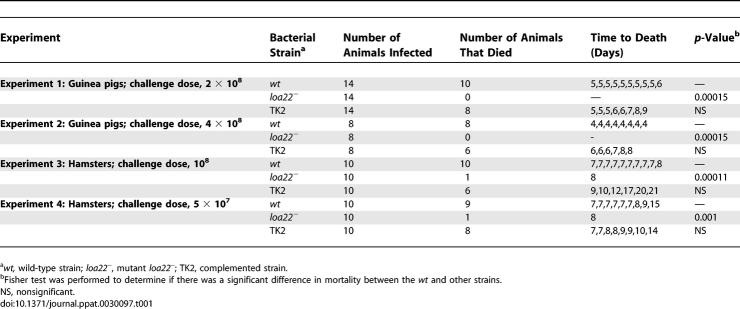
Virulence of L. interrogans Serovar Lai Strain Lai and Its Derivatives in the Guinea Pig and Hamster Infection Models

**Figure 3:**
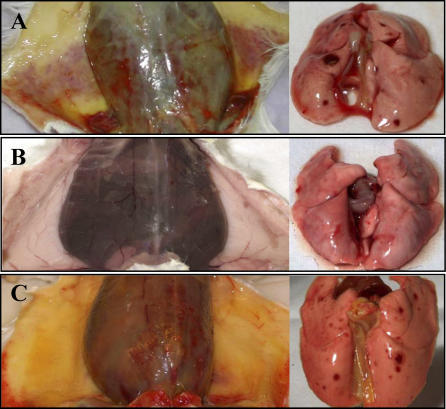
Gross Examination of Infected Guinea Pigs Left panel: Guinea pigs infected with the wild-type (A) and complemented strains (C) with clinical findings of jaundice and hemorrhages that are absent in guinea pigs infected with the mutant *loa22^−^* strain (B). Right panel: Lungs of a guinea pig infected with mutant *loa22^−^* did not exhibit macroscopic hemorrhage (B), in contrast with lungs of guinea pigs infected with the wild-type (A) and complemented strains (C). Tissues were observed 6 d post-inoculation.

In contrast, the mutant *loa22* ^−^ strain demonstrated loss of virulence, as reflected by the inability of challenge doses of 2 × 10^8^ and 4 × 10^8^ bacteria to produce death in guinea pigs (14 and eight animals, respectively) (Table 1). The difference in mortality was significantly lower for animals challenged with the *loa22* ^−^ than those challenged with the wild-type strains (0% versus 71% and 0% versus 100% in experiments 1 and 2, respectively, *p* < 0.05). Guinea pigs infected with the *loa22* ^−^ strain did not demonstrate clinical signs of leptospirosis during the 21-d follow-up period. The mutant *loa22* ^−^ strain was isolated from blood at post-challenge day 3 in four of four infected guinea pigs that were infected with 2 × 10^8^ bacteria in a separate experiment. In addition, the *loa22* ^−^ strain was isolated from the kidneys of five of seven guinea pigs killed at post-challenge day 21 (experiment 1, Table 1). However, cultures of kidneys from animals infected with the *loa22* ^−^ mutant required an incubation period of more than 2 wk to test positive for the bacteria, suggesting that the number of viable leptospires in these tissues was low. In addition, when cultures of livers of guinea pigs infected with the wild-type strain were positive for infection, we were not able to isolate the *loa22* ^−^ strain by culture of liver tissues from seven guinea pigs killed at post-challenge day 21. These findings indicated that although the mutant did not induce disease, it was able to cause bacteremia and colonization following infection. Sequential in vivo passaging and re-isolation of the *loa22* ^−^ strain from blood or tissues of infected guinea pigs (seven cycles in total) failed to recover a virulent isolate that could induce clinical disease or death in guinea pigs.

Complementation of *loa22^−^* restored the virulence phenotype of the mutant *loa22* ^−^ strain in the guinea pig infection model. Challenge doses of 2 × 10^8^ and 4 × 10^8^ of TK2 bacteria caused death in 43% and 75%, respectively, of the inoculated animals (Table 1). Deaths occurred 5 to 9 d after challenge. There were no significant differences between the death rates among guinea pig groups challenged with the wild-type and TK2 strains. DNA was extracted from TK2 strains that were used to challenge guinea pigs and TK2 strains that were re-isolated from guinea pigs during autopsy. Southern blot and PCR analyses demonstrated that these isolates had the complemented *loa22* genotype and the spectinomycin and kanamycin cassettes (unpublished data), indicating that the observed restoration in virulence was not due to contamination of inoculating cultures with the wild-type strain.

Hamsters were challenged with wild-type, mutant *loa22* ^−^, and TK2 strains to confirm the findings observed in the guinea pig model. Inoculation with 10^8^ and 5 × 10^7^ wild-type bacteria induced death in 100% and 90%, respectively, of the animals (Table 1, experiments 3 and 4, respectively). In contrast to what was observed in the guinea pig model, challenge with mutant *loa22* ^−^ bacteria caused death in one of ten hamsters in the two experiments. Autopsy evaluation performed in experiment 4 found that the hamster died from manifestations of leptospirosis. However, death rates were significantly lower (10% versus 100%, *p* = 0.00011; and 10% versus 90%, *p* = 0.001 for experiments 3 and 4, respectively) for hamsters challenged with *loa22* ^−^ than those challenged with wild-type strains. Challenge with the TK2 strain produced death in 60% (six of ten) and 80% (eight of ten) of the hamsters in experiments 3 and 4, respectively, indicating that as in the guinea pig model, complementation of *loa22* in the mutant strain partially restored virulence.

### Mutant *loa22^−^* Strain Does Not Produce Tissue Pathology in the Guinea Pig Model

Necropsy evaluation of guinea pigs infected with wild-type strain at post-challenge days 5 and 6 found macroscopic lesions associated with leptospirosis (Figure 3A). Diffuse hemorrhage was observed in kidneys, and multi-focal hemorrhage was seen in lungs, stomachs, and intestines (unpublished data). Splenomegaly was observed, as well as jaundice of the liver and subcutaneous, ascites, and hemothorax. None of these findings was observed, except for splenomegaly, in necropsies of guinea pigs infected with the mutant *loa22* ^−^ strain (Figure 3B). Infection with the TK2 strain, in which *loa22^−^* was complemented, produced the complete spectrum of gross lesions observed in infections with wild-type strain (Figure 3C).

Hematoxilin and eosin staining of sectioned lung, kidney, spleen, and liver from guinea pigs infected with the wild-type strain demonstrated characteristic histopathologic findings for leptospirosis (Figure 4). Spleens were hemorrhagic, with focal necrosis in the red pulp (unpublished data). Intra-alveolar hemorrhage associated with interstitial infiltration with polymorphonuclear and mononuclear cells was a prominent finding in lung sections (unpublished data).

**Figure 4:**
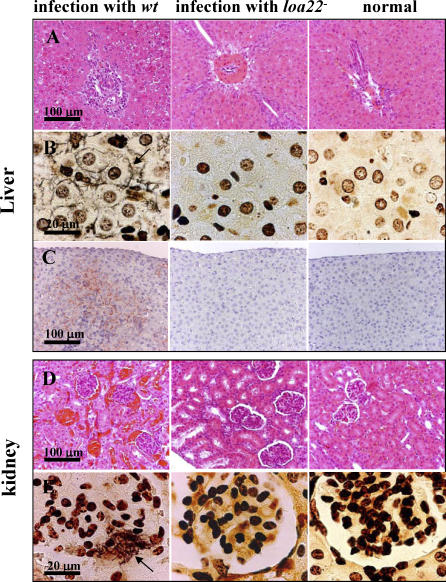
Livers and Kidneys from Guinea Pigs Infected with the Wild-Type Strain and Mutant *loa22^−^* Strain All images are from guinea pigs 6 d post-inoculation. The right panels show normal livers (A–C) and kidneys (D and E). Tissues were stained by hematoxylin and eosin (×200, [A and D]), Warthin–Starry (×1000, [B and E]) and immunohistochemistry with antiserum specific to LipL32 (×200, [C]). Left panel, wild-type strain *(wt);* middle panel, mutant *loa22^−^* strain. (A–C) Livers of *wt*-infected animals exhibit important periportal lymphoplasmocitary inflammatory infiltration, loss of parenchymal architecture, and increase of biliary canalicules in comparison with a normal aspect of mutant *loa22^−^*. Distortion of liver cords is related to numerous leptospires along cell membranes of hepatocytes (arrow, [B]) in *wt*-infected animals. (D and E) Kidneys of *wt*-infected animals present hemorrhages in Bowman's spaces, lumen of renal tubules, and interstitium (D). A large number of leptospires is seen in Bowman's space (arrow), sometimes forming a cap (E). Histology of kidneys infected with mutant *loa22^−^* was considered as normal (D and E).

However, infection with mutant *loa22^−^* strain produced markedly reduced or absent inflammatory responses and tissue pathology in guinea pigs on post-challenge day 6 (Figure 4). Liver tissues demonstrated mild parenchymal dystrabeculaton and periportal infiltrates without focal necrosis or hemorrhage (Figure 4A, middle panel). Kidneys, spleens, and lungs from mutant-infected animals exhibited sparse or absent inflammatory infiltrates. Infection with the TK2 strain, in which *loa22^−^* was complemented, produced similar pathological findings as observed for the wild-type strain (Figure 5).

**Figure 5:**
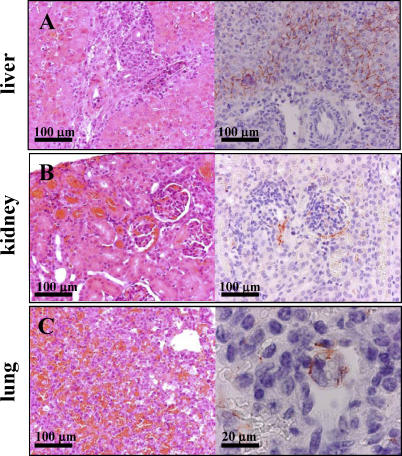
Histopathologic sections on Liver, Kidney, and Lung of Guinea Pigs Infected by Complemented Strain TK2 6 d Post-Inoculation Left panel: Hematoxylin and eosin staining (×200) of infected guinea pigs. Right panel: Immunochemistry with antiserum specific for LipL32 (×200; except [C], ×1,000). Pictures of histopathology were similar between animals infected with wild-type and complemented strains. (A) The liver has a great loss of architecture and areas of necrosis and inflammatory infiltration, which are both associated with the presence of numerous leptospires. (B) Kidneys present hemorrhages, tubular necrosis, and inflammatory infiltration, with leptospires mainly located in Bowman's spaces and proximal tubules. (C) Lungs have marked intra-alveolar hemorrhages with inflammatory infiltrates, and few leptospires are present within septal membranes and, sometimes, in macrophages ([C], right panel).

Silver staining and immunohistochemistry demonstrated the abundant numbers of leptospires in the livers (Figure 4B and 4C, left panel) and kidneys (Figure 4E, left panel) of guinea pigs infected with the wild-type strain at post-challenge day 6. Sparse numbers of leptospires were found in the interstitial and alveolar spaces of the lungs. In contrast, leptospires were not detected in tissues of guinea pigs infected with the *loa22^−^* strain at post-challenge days 6 (Figure 4B, 4C, and 4E, middle panel) and 21. In sectioned kidneys and livers from guinea pigs infected with the complemented TK2 strain (Figure 5), immunohistochemical analyses identified leptospires in numbers similar to those observed for wild-type infections. Antiserum to Loa22 stained all wild-type (Figure 6) and TK2 (unpublished data) leptospires found in kidney and liver sections, demonstrating that this protein is expressed during acute leptospirosis.

**Figure 6:**
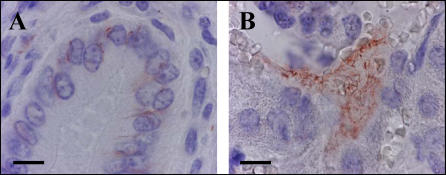
In Vivo Expression of Loa22 in Liver and Kidney of Guinea Pigs Infected with L. interrogans Serovar Lai (A) Liver, (B) kidney. Histopathologic sections were stained by immunochemistry using Loa22 antiserum (×1,000). Intact organisms were found in biliary ducts (A) and in large number in Bowman's spaces and proximal tubules (B). Scale bar = 20 μm.

## Discussion

The recent completion of the genome sequences of pathogenic *Leptospira* strains has led to the identification of putative determinants that may play a role in virulence [9–11]. One such determinant, *loa22,* is up-regulated during host infection [17] and encodes a lipoprotein with an OmpA domain [14] that is strongly recognized by sera from human leptospirosis patients [15]. Furthermore, Loa22 is conserved among pathogenic *Leptospira* [14–16], suggesting that it may play a specific role in disease pathogenesis. However, its role has not been elucidated until now, because targeted mutagenesis was not previously feasible in pathogenic *Leptospira*. Recently, we showed that the *Himar1 mariner* transposon permits random mutagenesis in the pathogen L. interrogans [13]. In search of mutants that might be affected in virulence, we identified an L. interrogans mutant exhibiting *Himar1* insertion into *loa22*. By analysis of the *loa22^−^* strain, we now show that Loa22 is required for virulence of the pathogen within animal models and fulfills the molecular Koch's postulates [12] as a virulence factor.

Complementation of the virulence phenotype of the *loa22^−^* strain by chromosome insertion of *loa22* demonstrated that the virulence defect was due to the inactivation of *loa22* and not to a second-site mutation. Transcriptional data and sequence analysis of the transposon insertion sites in the mutant and complemented strains further confirm that *Himar1* insertion did not affect another gene that could be involved in virulence (unpublished data). The parental and mutant strains of L. interrogans showed similar cell morphology and growth characteristics in vitro, which demonstrates that *loa22* is not essential for in vitro growth. Although there was no statistical difference in death rates among animals challenged with wild-type and complemented strains, infections with the complemented strain did not cause death in all animals. The inability to restore complete virulence (100% lethality) in the complemented strain did not appear to be due to instability of the construct, because the complemented strain resulted from chromosome insertion. The complemented strain that was re-isolated from animals expressed Loa22. In addition, infection with another strain, TK1, for which the *loa22* gene was complemented at another chromosomal site, caused death rates in guinea pigs (six of eight guinea pigs; challenge dose: 4 × 10^8^ bacteria/ml ), which was not significantly different from results obtained for infections with the TK2 strain (Table 1). The complemented strains were subjected to more in vitro passages than the wild-type strain due to electroporation-mediated transformation followed by plating onto solid medium. Leptospires are known to lose their virulence phenotype with prolonged in vitro culture passages [21], although the mechanism for this loss is not well understood. It is also possible that the complemented *loa22* gene did not attain the optimal level of expression required for the virulence phenotype.

Infection with L. interrogans produces a lethal infection in the standard hamster and guinea pig model and mimics the clinical presentation of severe leptospirosis in humans (i.e., jaundice and pulmonary hemorrhage) [1–3]. Loss of *loa22* attenuated the ability of leptospires to cause clinical disease in addition to death in guinea pigs and hamsters. Consistent with the lack of disease manifestations, mildly abnormal or no pathologic changes were observed in tissues of guinea pigs infected with the *loa22^−^* strain. Although the *loa22* ^−^ strain was recovered by culture isolation from blood on post-challenge day 3 and in kidneys of guinea pigs during the 21-d post-inoculation period, immunohistochemical analysis did not detect leptospires in these tissues, suggesting that loss of the *loa22* genotype reduced the pathogen burden in tissues during infection. The lack of tissue pathology observed in *loa22* ^−^-infected animals presumably reflects decreased inflammation elicited by lower numbers of leptospires. Loa22 may influence one of several virulence steps during infection, such as the ability to disseminate throughout the host after inoculation, adhere to host cells, and establish persistent infection, which may, in turn, explain the finding that mutant *loa22* ^−^ strain did not achieve sufficient numbers in tissues to produce disease manifestations and pathology. The standard inoculation method used in animal models of leptospirosis—intraperitoneal injection—may not reflect conditions encountered during natural infection, because leptospires enter the host by penetrating breaks in the skin or traversing the mucosal membranes. Further studies that use subconjunctival or subcutaneous challenge routes will need to be performed to determine whether Loa22 plays a role in the initial steps of infection.

The process of host infection by pathogens is usually complex and multifactorial. We observed that loss of *loa22* genotype was associated with complete loss of virulence in the guinea pig model, as indicated by the inability to induce death in these animals. In hamsters, infection with the mutant *loa22^−^* strain was associated with significantly reduced death rates (10% versus 100%, mutant versus wild-type strain, respectively) but did not lead to complete loss in the ability to produce a lethal infection. The observed differences may reflect intrinsic differences in susceptibility of the two animal models to infection with the strain L. interrogans serovar Lai, which was used in this study.

Our results demonstrate that Loa22 is exposed on the surface of *L. interrogans,* confirming the localization of the protein to the outer membrane [14]. The structure of Loa22 is composed of a C-terminal OmpA domain of approximately 110 amino acids. This OmpA domain refers to the C terminus of Escherichia coli OmpA, a major outer membrane protein of E. coli. Orthologs of the OmpA domain are found in proteins from a wide range of bacterial species. Predictions for the structure of this C-terminal OmpA domain have ranged from that of a globular domain containing a predicted peptidoglycan-associating motif that is located in the periplasm [22,23], to a domain containing a significant proportion of anti-parallel β sheets that are associated with the outer membrane [24,25]. The N-terminal domain of E. coli OmpA was crystallized as a β-barrel–structured porin, which is believed to be inserted into the outer membrane [26]. However, this N-terminal region has no significant sequence similarity to Loa22. Because there is no sequence similarity between Loa22 and other OmpA-like proteins in this N-terminal region, these proteins may be structurally distinct.

The role of Loa22 during pathogenesis remains to be determined. The OmpA protein of E. coli and other Gram-negative bacteria are believed to play a multifunctional role in bacterial physiology and pathogenesis. In Gram-negative bacteria, OmpA has been shown to be an adhesin [27–29] and to induce cytokine production by dendritic cells [30,31]. In a recent study, recombinant Loa22 was shown to bind in vitro to a limited extent with components of the extracellular matrix such as plasma fibronectin and collagen types I and IV [5], suggesting that the surface-exposed domain of Loa22 may, in fact, act as an adhesin. Furthermore, the lipopeptide moieties of spirochetes are potent mediators of the inflammatory response [8]. Loa22, which has a lipobox sequence, may therefore induce severe disease manifestations by eliciting the host immunopathogenic responses.

Proteins of the OmpA family have been proposed to play a role in the stabilization of the envelope structure [23]. Loa22 , which includes a predicted peptidoglycan-associating motif in its C-terminal domain [14], may form a bridge linking the protoplasmic cylinder, including the peptidoglycan layer, and the outer membrane. Although the *loa22^−^* strain was recovered from the animal, we cannot rule out that the absence of this protein in the membrane may affect several steps in host infection, such as the stability of the outer membrane, survival of the leptospiral pathogen in vivo, and the ability to penetrate tissues during dissemination or adhere during colonization.

In conclusion, this study identified the first virulence factor, to our knowledge, in pathogenic *Leptospira* and will form the basis for further investigation of the role that Loa22 plays in leptospiral pathogenesis. Furthermore, Loa22 is expressed on the leptospiral surface, suggesting that immunization with this protein may elicit bacteriocidal or pathogenesis-blocking immune responses. Bacterin-based vaccines have been used in some countries but they present a number of disadvantages, including adverse reactions, short duration of efficacy, and lack of protection against serovars not included in the vaccine preparations [1–3]. A better understanding of the role of Loa22 may facilitate identification of defined and more effective subunit vaccine candidates for leptospirosis.

## Materials and Methods

### Bacterial strains and growth conditions.

L. interrogans serovar Lai strain Lai 56601 (gift from the National Institute for Communicable Disease Control and Prevention, ICDC China CDC) and other *Leptospira* strains were grown at 30 °C in EMJH [32,33] liquid medium or on 1% agar plates. E. coli was grown in Luria-Bertani (LB) medium. When necessary, spectinomycin or kanamycin was added to culture media at 50 μg/ml.

### Construction of mutant and complemented strains.

Random insertion mutagenesis was carried out in low-passage L. interrogans serovar Lai strain Lai 56601 with plasmid pMSL as previously described [13]. After 4 to 6 wk of incubation, spectinomycin-resistant transformants were inoculated in liquid medium. Genomic DNA was then extracted, and the transposon insertion site of each transformant was identified by ligation-mediated PCR as previously described [34]. Among the transformants, we selected a mutant with an insertion into *loa22,* also called LA0222, at position 220548 in the large chromosome (CI) of L. interrogans for further characterization. For complementation, *loa22* was amplified with primers OMIA (5′-AGTCGACGGTTTTGGTGGGATGGATAG-3′) and OMIB (5′-AGTCGACAGACGTTGAGTTGCCACAGC-3′) and cloned into the SalI restriction site of the kanamycin-resistant transposon carried by plasmid pMKL, resulting in plasmid pMKLoa22. The mutant *loa22* ^−^ strain was then electrotransformed by pMKLoa22, and the transposon insertion site of some transformants was identified as described above. Two transformants, strains TK1 and TK2, were further studied; one, strain TK1, exhibited the kanamycin-resistant transposon at position 1079614 (between LA1074 and LA1075), and the other, strain TK2, at position 84051 (into LA0071) of the large chromosome of L. interrogans. Confirmation of genotypes was performed by using PCR with primers S1a (5′-TTGTTGTGGTGCGGAAGTCG-3′) and S1b (5′-GGTCCCGAACAAGCAGAAGG-3′), which are located in the flanking sequences of the transposon inserted into *loa22,* and Southern blots.

### Enzyme-linked immmunosorbent assay (ELISA).

L. interrogans strains were grown in EMJH until the culture reaches an optical density at 420 nm (OD_420_) of 0.4. L. biflexa was also used a control. Concentrations were adjusted to 10^9^ bacteria/ml in a volume of 20 ml of EMJH, and 40 μl of 37% formaldehyde was added, then incubated 2 h at room temperature and boiled for 30 min. After adjusting pH at 9.6, cultures were centrifuged at 8,000*g* for 20 min and pellets were resuspended in 10 ml of 0.05 M bicarbonate buffer. Ninety-six–well flat-bottom polystyrene assay plates (Immulon, VWR, http://www.vwr.com/) were coated overnight at 4 °C with 50 μl of total bacterial antigen.

Plates were washed three times with phosphate buffered saline (PBS) (pH 7.2) and wells were blocked with 50 μl of 5% nonfat milk PBS for 45 min at 37 °C. Plates were incubated 45 min at 37 °C with 50 μl of an 800-fold dilution of mouse polyclonal antiserum to Loa22 [14] diluted in milk PBS, washed, and incubated for 1 h at 37 °C with 50 μl of a 2,500-fold dilution of horseradish peroxidase–conjugated sheep affinity–purified antibody specific to mouse immunoglobulin G (IgG) (Promega, http://www.promega.com/). After washing of the plates, 50 μL of ABTS peroxidase substrate (Roche, http://www.roche.com/) was added, and the plates were incubated in the dark at room temperature for 25 min. Optical density was measured using an ELISA reader (Labsystems Multiskan MS; Thermo Scientific, http://www.thermo.com/) at 405 nm.

### Localization of Loa22 by immunofluorescence.

Surface immunofluorescence labeling was performed according to a modified protocol of Cullen et al. [35]. Suspensions of 10^7^ live leptospires in 10 μl of PBS were placed onto poly-L-lysine–coated (Sigma, http://www.sigmaaldrich.com/) slides for 1 h in a humidified chamber. The slides were washed twice with PBS with 2% bovine serum albumin (PBS-BSA) and were incubated for 1 h with antisera (diluted 1:100 in PBS-BSA) to recombinant *Leptospira* proteins. After incubation with mouse antiserum to Loa22 [14] and rat antisera to LipL32, LipL41, LipL31, and GroEL, the slides were washed gently with PBS-BSA. Leptospires were fixed by applying cold methanol and incubating the slides for 10 min at −20 °C. The slides were then washed and incubated with donkey anti-mouse IgG antibodies conjugated to Alexa dye (Molecular Probes, http://probes.invitrogen.com/) or goat anti-rat IgG antibodies conjugated to fluorescein isothiocyanate (Jackson ImmunoResearch Laboratories, http://www.jacksonimmuno.com/) for 1 h at 37 °C. The slides were washed twice with PBS-BSA and incubated with 1 μg/ml DAPI (Molecular Probes) for 1 h at room temperature. The slides were mounted in anti-fading solution after washing and before visualization of stained organisms with fluorescence microscopy.

### Animal infections.

Golden Syrian male hamsters, 5 to 8 wk old, and Hartley male guinea pigs (Charles River Laboratories, http://www.criver.com/), 2 to 3wk old, were used for this study. Animals were maintained under standard conditions according to institutional guidelines. Water and food were given ad libitum. All animal infections were performed with intraperitoneal injection of low-passage strains in 1 ml of EMJH medium. Negative control animals were injected intraperitoneally with 1 ml of EMJH medium. Animals were monitored daily for characteristic signs of leptospirosis (i.e., prostation and jaundice) and survival. Surviving animals were killed after a 21-d post-challenge follow-up period. The 50% lethal dose (LD_50_) for L. interrogans serovar Lai in 2- to 3-wk-old guinea pigs and 5- to 8-wk-old hamsters was approximately 10^8^ and 10^7^ leptospires, respectively. Protocols for animal experiments were prepared according to the guidelines of the Animal Care and Use Committees of the Institut Pasteur and Fundação Oswaldo Cruz.

### Histopathology.

Guinea pigs were inoculated with 2 × 10^8^ bacteria of wild-type, mutant *loa22* ^−^, and complemented strains of L. interrogans serovar Lai strain Lai or EMJH alone. For mutant *loa22* ^−^ strain and EMJH control group infections, three guinea pigs were killed 6 and 21 d post-inoculation. For wild-type and TK2 strain group infections, tissues were collected at the day of death (5 or 6 d post-inoculation). Tissues (liver, kidneys, spleens, and lungs) were fixed in 10% buffered formaldehyde, embedded in paraffin, and sectioned according to routine histological procedures to produce 5-μm sections that were then stained with hematoxylin and eosin and Warthin–Starry silver impregnation [36]. For immunohistochemistry, paraffin was removed from the sections with xylene and ethanol. Tissues were then treated in citrate buffer (pH 6) at 98 °C for 1 h and nonspecific sites were blocked by incubation of sections with 1.5% BSA at room temperature for 20 min. Tissues were incubated with 6,000- and 1,000-fold dilution of LipL32 [37] and Loa22 [14] antisera, respectively, overnight at 4 °C. Samples were treated with 0.3% hydrogen peroxide for 30 min at room temperature, then incubated at room temperature for 30 min with goat anti-mouse or anti-rabbit antibodies conjugated to peroxidase (Dako Cytomation, http://www.dako.com/). Enzyme reactions were developed using AEC (3-Amino-9-ethylcarbazole) staining kit (Sigma). The pathologist viewed the histopathological preparations without knowing the infection status of the animals.

## Supporting Information

### Accession Numbers

The Entrez Genome (http://www.ncbi.nlm.nih.gov/sites/entrez?db=Genome) accession numbers for the genes and gene products discussed in this paper are *L. borgpetersenii* serovar Hardjobovis (NC_008508 and CP000348), L. interrogans serovar Copenhageni strain Fiocruz L1–130 (AE016823), and L. interrogans serovar Lai strain Lai 56601 (NC_004342).

## Abbreviation

ELISA: enzyme-linked immunosorbent assay
